# Is temperature change a key driver of the regional differences in electricity consumption of the economic sectors in Spain (2000–2016)?

**DOI:** 10.1007/s11356-023-27789-2

**Published:** 2023-06-14

**Authors:** Rocío Román-Collado, María J. Colinet-Carmona, María I. Fárez-Plasencia

**Affiliations:** 1 Universidad de Sevilla, Seville, Spain; 2 Universidad Autónoma de Chile, Providencia, Chile; 3 Departamento de Análisis Económico y Economía Política, Facultad de Ciencias Económicas y Empresariales, Avda. Ramón y Cajal, s/n, 41018 Seville, Spain; 4 Agencia Andaluza de la Energía, Seville, Spain; 5 Universidad de Cuenca, Cuenca, Ecuador

**Keywords:** Electricity consumption, Economic sectors, Spatial-temporal decomposition analysis, Temperature changes, Spain

## Abstract

Climate change has caused significant changes in temperature with different consequences depending on the geographical location of the regions, affecting among other aspects, electricity consumption (EC). Spain being a country that encompasses so many different temperature zones, this work analyses EC per capita among the Autonomous Communities (AC) of Spain through a spatial-temporal decomposition analysis during the 2000–2016 period. The regional differences are explained by four decomposition factors: intensity, temperature, structural and per capita income. The temporal decomposition results show that temperature changes in Spain between 2000 and 2016 have substantially affected the per capita EC. Likewise, it has been noted that in the 2000–2008 period, the temperature effect mainly acted as an inhibitor compared to the 2008–2016 period, in which an increase in the days of extreme temperature acted as a driver. The spatial decomposition reveals that the structural and energy intensity effects contribute to the AC moving away from average figures, while the temperature and income effects contributes to reducing the differences depending on the location of the AC. The results enable to determine the importance of establishing public policy measures aimed at improving energy efficiency.

## Introduction

According to the Fifth Assessment Report of the Intergovernmental Panel on Climate Change, the average global surface temperature has increased by 0.85 °C (ranging between 0.65 and 1.06 °C) between 1880 and 2012 and will probably rise by 1.5 °C (ranging between 0.3 and 4.8 °C) by the end of the twenty-first century (IPCC 2013). In Europe, the warmest summer temperatures over the last 2000 years have been recorded in recent decades (Luterbacher et al. 2016). The forecasts of the European Environment Agency (EEA 2017) show that climate change can cause seasonal changes in energy consumption in Europe with large regional differences.

Specifically, the temperature changes stemming from the consequences of climate change have a major impact on electricity consumption (EC) (Climent et al. 2003; Pardo et al. 2002; Valor et al. 2001; Zhang et al. 2019a; Zhang et al. 2021). Due to its impact on demand for heating and cooling, most of the existing studies analyse the relationship between climate change and EC focussing on the residential sector. In the case of Shanghai, the analysis conducted by Yi-Ling et al. (2014) shows that winter and summer are the two peak periods in demand for electricity due to the urban residential demand for heating and cooling. For the case of Jiangsu in China, Zhang et al. (2020) show that both a higher demand in cooling in summer and a higher demand in heating in winter lead to higher EC. More specifically, Hou et al. (2022) highlighted that the largest increase in electricity demand will occur in August considering that temperatures above 34 °C will increase residential demand for electricity monthly by 2.11%. Due to warmer temperatures, Li et al. (2018) show that the electricity demand in winter decreases while it increases in summer due to refrigeration and air conditioning. Examples, such as the analysis of Zheng et al. (2020) for the case of Guagzhou, identify an increase in demand for cooling and EC caused by a rise in temperatures. Also, Hou et al. (2022) highlight the fact that the increase in temperatures due to global warming will mainly affect the urban electricity consumption of low-income groups due to the expected increase in demand. In the case of residential demand, the analyses of Zhang et al. (2021) show that although income growth has a positive effect on demand for electricity, it also acts as a crowding-out effect since residents might look for the most efficient electricity appliances.

In addition to residential, changes in EC due to climate change also affect other economic sectors. However, there are few research analyses on this topic. Focusing on Singapore and Hong Kong, Ang et al. (2017) determine that increases in EC in relation to temperature are the highest in the residential sector, followed by the commercial and industrial sectors. For the residential and commercial sector, Zachariadis and Pashourtidou (2007) in their study for Cyprus find that climate fluctuations are the most important cause of short-term variation in EC; Lam et al. (2009), focusing on Hong Kong, reveal that EC in the residential sector increases in summer due to the use of air-conditioning, while it also increases in the commercial sector but over a longer period of the summer; Hong et al. (2013) and Zhou et al. (2014) analyse the residential and commercial sector in the USA through the use of energy by buildings in these sectors, with their results indicating that climate change has a major impact on the use of energy and fuel by buildings for heating and cooling.

Furthermore, the impact of climate change on EC has also been analysed from a regional and national perspective in several studies considering the difference in temperature between the northern and southern regions. Bessec and Fouquau (2008) use monthly aggregate EC data for 15 European countries, with their results indicating that the temperature effect on consumption is more pronounced in warmer countries. Following this approach, and analysing the case of Southern European countries, Silva et al. (2020) show that extreme temperatures and weather events are those that can have an important impact on EC. Therefore, countries with a milder weather will be less affected. Ahmed et al. (2012) establish that aggregate electricity demand in summer and spring will increase due to climate change in the Australian state of New South Wales, leading to an 11.3% increase in spring 2100. Focusing on Italy, Apadula et al. (2012) analyse the effect of weather conditions on aggregate monthly electricity demand and find that temperature is the most important variable for forecasting electricity demand. Spain is another study case by Garrido-Pérez et al. (2021), concluding that the frequency and severity of days with extreme demand for electricity is expected to increase, although there is considerable spatial heterogeneity throughout the country.

In the EU-28, the average EC has grown by 9% between 2000 and 2016, going from 94,025 GWh to 102,603 GWh. In fact, 2016 registered a rise of 2.7% with respect to 2015, after 4 years of declining EC. Throughout the 2000–2016 period, Spain has been one of the top five EU-28 countries in terms of EC, only below Germany, France, the UK, and Italy (EUROSTAT 2020). Specifically, EC rose by 23% in Spain between 2000 and 2016, reaching 245,769 GWh in 2016. Although Spain has improved its energy efficiency by 1.44% annually between 2000 and 2016 according to the energy efficiency index used in the ODYSSEE-MURE project (ODEX) and achieved energy savings of 15.8% in 2016, surpassing the 9% goal set for that year in Directive 2006/32/EC (IDAE 2018), it has not managed to reduce EC in absolute terms.

Spain faces a significant challenge that does not only involve energy, it also concerns the climate. On the one hand, Spain must reduce EC to achieve its energy saving targets and energy efficiency in order to fulfil the objectives set by the European Union which involves a 32.5% reduction in EC by 2030 (European Union 2018). This latter aim will contribute to the decarbonisation of the Spanish economy by 2050 (European Union 2019). However, there are climate factors such as the temperature changes and the diversity of climate zones which can affect this challenge (López-Ochoa et al. 2021).

In relation to the first issue, EC accounts for 23% of the total energy consumed in Spain and is the second largest energy source consumed after petroleum products, which represented 53% of total consumption in 2019 (Ministerio para la Transición Ecológica 2019). Along with the residential sector, the key economic sectors that explain EC in Spain are the service and industrial sectors and, to a lesser extent, the transport sector. EC is the most relevant in the case of the service sector (62% of the total consumption in 2017) which is used in lighting, air-conditioning, office equipment, and information and communication technology (ICT). The service sector makes up a significant share of the Spanish production structure (66% of GDP), with a high dependence on EC (13 percentage points above the EU average). Despite of the fact that the consumption of fossil fuel sources (coal, oil, and natural gas) for heating in predominant in the industrial sector, EC went from 29 to 35% between 2000 and 2017. In addition, its industrial subsectors, such as non-metallic minerals and metallurgy, have a high energy intensity, above the European average (Ministerio para la Transición Ecológica 2019). As for the transport sector, the consumption of petroleum products accounts for the bulk of final energy consumption (94%), compared to 6% for alternative energy consumption, i.e., EC was only 21% of alternative energy in 2017 (Ministerio para la Transición Ecológica 2019). The importance of the rebound effect caused by energy efficiency has been explored in the case of Spain, with concerns raised over the industry, transport and service sectors (Cansino et al. 2019). These elements undoubtedly influence EC in Spain and in its different regions or Autonomous Communities (AC), given the differences in this structure among the AC.

The climate in Spain is extremely diverse due to its geographical location, and temperatures show a wide range between the minimum and the maximum values. Additionally, it should be considered that the anomalous temperature variations stemming from climate change will not affect all AC similarly because they are located in different climate zones. Specifically, from the perspective of the technical building code, there are 15 climate zones in Spain based on the climate’s severity in winter and summer, showing the evolution of the climate zoning of all Spanish provincial capitals and autonomous cities (López-Ochoa et al. 2021; Las-Heras-Casas et al. 2021). AEMET and IPMA (2011) identify five climates in Spain (Mediterranean, Semiarid, Continental, Oceanic, and Subtropical), showing significant climate fluctuations, with temperatures below 0.0 °C in high altitude areas in winter and temperatures above 27 °C in certain areas in summer (AEMET and IPMA 2011). According to AEMET (2019), the average summer temperature from 1971 to 2018 has increased in Spain both in frequency and intensity, due to climate change. Higher average temperatures and more extreme maximums and minimums have also been recorded, with many of the historic extremes of maximum temperatures concentrated in the last decade (Serrano-Notivoli et al. 2022). The literature review reveals the scientific interest in the relationship between EC and temperature in Spain. Specifically, Pilli-Sihvola et al. (2010) find that electricity demand in Spain is set to increase by 2–4% by 2050, a projection based on 2007 data. Additionally, Díaz-López et al. (2021) show that it is expected that by the year 2085, practically all cities in Spain will change their climate zone to warmer ones, increasing the demand for cooling.

Decomposition techniques have been widely used to analyse and better understand the behaviour of energy and pollutant emission variables (Wang et al. 2017). In Spain, the logarithmic mean Divisia index (LMDI) analysis has been used to analyse the determinants of changes in energy consumption (Román-Collado and Colinet 2018), CO_2_ emissions in the 1995–2009 period (Cansino et al. 2015), and energy efficiency (Mendiluce et al. 2010; Mendiluce 2007, 2012), while Román-Collado and Colinet (2018) combine the index decomposition analysis (IDA) and the structural decomposition analysis (SDA) approaches to analyse the energy consumption of the economic sectors and households in Spain. Other studies focus on a specific region. For example, Colinet and Román-Collado (2016) analyse the changes in final energy consumption in Andalusia between 2002 and 2013, and López-González et al. (2018) evaluate final energy consumption, primary energy consumption and the contribution of renewable energies in the residential sector in La Rioja. Given the weight of certain sectors in the production structure or in energy consumption, some studies for Spain focus their analysis on a specific sector: Arocena et al. (2016), Boyd and Roop (2004), and Fernández-González and Pérez-Suárez (2003) analyse the drivers of the observed change in the energy intensity of Spanish manufacturing industries; Sobrino and Monzon (2014) and Andrés and Padilla (2015) assess changes in energy intensity in the road freight transport sector; and Fernández-González and Moreno (2015) analyse the drivers of EC in the electricity sector.

A variant of the abovementioned LMDI decomposition analyses is the spatial version introduced by Ang et al. (2015). This method allows the researcher to compare differences in energy consumption or CO_2_ emissions between regions within a country (or between countries with harmonised data) in a particular year. Studies such as those by Chen et al. (2019), Li et al. (2017), and Liu et al. (2018) use this method to analyse carbon intensity in China at provincial and regional level; and Román-Collado and Morales-Carrión (2018) analyse the differences in CO_2_ emissions for Latin American countries. However, given the static nature of these studies, Ang et al. (2016) developed a procedure known as the spatial-temporal index decomposition analysis (ST-IDA) that simultaneously integrates the analysis of spatial differences and the study of the temporal behaviour of regions and/or countries for environmental variables. The majority of the studies which have used this methodology have done so for the provinces and regions of China to analyse the regional differences in water or electricity consumption and CO_2_ emissions. Wang et al. (2018) and Yang et al. (2019) use a spatial-temporal analysis to determine the driving forces behind the regional differences in carbon intensity. Song et al. (2019) also study carbon intensity using ST-IDA but include an analysis of spatial agglomeration, which involves grouping the provinces into regions. Liu et al. (2018) analyse the drivers of energy consumption and CO_2_ emissions for the cement industry. Fang et al. (2019) use the ST-IDA model to decompose the growth in EC between 1995 and 2016 into four factors. Hang et al. (2019) use the ST-IDA to evaluate the regional differences in the reduction of SO_2_ emissions, while Yao et al. (2019) apply it to study the regional intensity of water consumption for the Yangtze River economic area. Román-Collado and Colinet (2021) use this approach for Andalusia (an AC of Spain) in comparison to Spain showing that the positive differences in energy consumption are mainly explained by its higher energy intensity.

The aim of this paper is to analyse the explanatory factors of the changes in EC of economic sectors in Spain throughout the period going from 2000 to 2016, considering changes in temperature among other factors. This study uses the spacio-temporal index decomposition analysis through the logarithmic mean Divisia index (ST-LMDI), applying it to per capita EC with a view to capturing the key drivers of spatial differences between AC and the temporal changes that occurred over the 2000–2016 period. This approach will allow to identify the influence that the differences in temperatures among the northern and southern regions besides the sector’s structure, the income, or the energy intensity might have on EC of economic sectors and how the changes on these factors have affected the EC of economic sectors along the analysed period. Based on the results of this research, conclusions can be drawn and recommendations can be made that are of interest not only to the academic community but also to policymakers. Policymakers can have information about how economic sectors are affected by temperature changes considering their dependency on EC, their energy efficiency and also their different relative weight in the gross value added (GVA) of each AC. This information might contribute to focus the energy policy and instruments to the appropriate sector and region.

The novelty of this research is twofold. The literature review has shown that the EC of residential sector has been affected by the temperature increase and somehow, income growth has contributed to counterbalance this effect by using more efficient electric appliances. However, a similar analysis has not been conducted for the other economic sectors. This is the reason why this research is focused on economic sectors in order to measure how the temperature increase affect their EC and how the different relative weight of economic sectors among AC might condition the energy savings and efficiency of regional economies. The second novelty of the paper is the methodological approach. The present study selects the ST-LMDI model, incorporating the temperature variable through an indicator showing the exceptional days with very low and high temperatures, along with other conventional effects such as income, structural, and population effects in order to identify the influence on the regional differences in Spain in EC per capita of economic sectors between 2000 and 2016.

The article is organised as follows. After this introduction, the method and materials are detailed in the “Materials and method” section. The main results are presented in the “Results” section, and they are discussed in the “Discussion” section. Finally, the main conclusions and recommendations are set out in the “Conclusions and policy implications” section.

## Materials and method

### Method

Following Ang et al. (2016), a spatial-temporal decomposition approach is conducted of the total EC per capita of *k* regions, specifically the 17 AC in Spain. The multiplicative model, unlike the additive model, has the advantage of considering the size of the economy under analysis, so its results are easily comparable among the different regions. Another important aspect is that, like the additive model, it has the additive property in logarithmic form.

The EC corresponding to the economic sectors of each Spanish region is decomposed into four factors as follows:

1 $${EEpc}^{kt}=\frac{EE^{kt}}{P^{kt}}=\sum_i\frac{EE_i^{kt}}{P^{kt}}=\sum_i\frac{\frac{EE_i^{kt}}{\frac{h^{kt}}{H^{kt}}}}{VAB_i^{kt}}\cdot\frac{h^{kt}}{H^k}\cdot\frac{VAB_i^{kt}}{VAB^{kt}}\cdot\frac{VAB^{kt}}{P^{kt}}=\sum\nolimits_i\frac{EE_{i\_ref}^{kt}}{VAB_i^{kt}}\cdot\frac{h^{kt}}{H^{kt}}\cdot\frac{VAB_i^{kt}}{VAB^{kt}}\cdot\frac{VAB^{kt}}{P^{kt}}$$

From, k=1,2,3,…,17 Spanish regions, i=1,..n, represents the economic sectors under analysis, specifically, the primary sector, manufacturing industries, non-manufacturing industries, construction, financial activities, public administration, and trade.

*EEpc* ^*kt*^ is the EC per capita of region *k* in year *t*, *EE*^*kt*^ is the EC of region *k* in year *t*; ${EE}_i^{kt}$ is the EC of region *k* in sector *i* in year *t*; *h* is the total number of days with temperatures lower than 0 °C and above 30 °C in region *k* in year *t*; *H* is the average number of days with temperatures lower than 0 °C and over 30 °C throughout the entire period (2000–2016) in region *k*; ${VAB}_i^{kt}$ is the GVA of sector *i* in region *k* in year *t*; *VAB*^*kt*^ is the total GVA of region *k* in year *t*; and *P*^*kt*^ is the total population of region *k* in year *t*. The expression ${EE}_{i\_ ref}^{kt}$ represents the EC requirements of sector *i* in region *k*, used exclusively for the economic activity of the economic sectors, when subtracting the changes in the EC due to the effect caused by the temperatures. This is done via climate correction (Economidou and Román-Collado 2019; Tsemekidi-Tzeiranaki et al. 2019).

In Eq. (1), the per capita EC of region k can be decomposed into four factors: energy intensity (*I*^*kt*^), temperature (*T*^*kt*^), structural (*S*^*kt*^), and per capita income (*Y*^*kt*^) as shown in the following equation:

2 $${EEpc}^{kt}=I^{kt}\cdot T^{kt}\cdot S^{kt}\cdot Y^{kt}$$

By applying the ST-LMDI model, the behaviour of the per capita EC in the different Spanish regions can be compared with a reference region throughout the considered period.

Firstly, according to Ang et al. (2016, 2015), the reference region has been constructed by calculating, for each variable, the arithmetic average of the Spanish regions in the last year of the period under analysis. This enables an analysis of how the Spanish regions stand with respect to the prior situation. For the reference region in 2016, the average per capita EC of the economic sectors has been calculated at 3.55 MWh/inhabitant.

Therefore, the factoring of the per capita EC for the reference region (*μ*) is:

3 $${ EE pc}^{\mu }=\frac{EE^{\mu }}{P^{\mu }}=\sum_i\frac{EE_i^{\mu }}{P^{\mu }}=\sum_i\frac{\frac{EE_i^{\mu }}{\frac{h^{\mu }}{H^{\mu }}}}{VAB_i^{\mu }}\cdot \frac{h^{\mu }}{H^{\mu }}\cdot \frac{VAB_i^{\mu }}{VAB^{\mu }}\cdot \frac{VAB^{\mu }}{P^{\mu }}={\sum}_i\frac{EE_{i\_ ref}^{\mu }}{VAB_i^{\mu }}\cdot \frac{h^{\mu }}{H^{\mu }}\cdot \frac{VAB_i^{\mu }}{VAB^{\mu }}\cdot \frac{VAB^{\mu }}{P^{\mu }}$$

4 $${EEpc}^\mu=I^\mu\cdot T^\mu\cdot S^\mu\cdot Y^\mu$$

with *I*^*μ*^ being the energy intensity, *T*^*μ*^ the temperature, *S*^*μ*^ the structural, and *Y*^*μ*^ the per capita income of the reference region.

According to Ang et al. (2015), the relative difference between the EC per capita of region *k* and the reference region (*u*) for a particular period (DEE) can be expressed by applying the spatial multiplicative LMDI method as follows:

5 $${DEE}^{kt-\mu}=\frac{EEpc^{k,t}}{EEpc^u}=D_I^{kt,u}\cdot D_T^{kt,u}\cdot D_S^{kt,u}\cdot D_Y^{kt,u}$$

where ${D}_I^{kt,u},{D}_T^{kt,u},{D}_S^{kt,u},{D}_Y^{kt,u}$ represent the intensity, temperature, structural, and income effect, respectively, and are defined as follows:

${D}_I^{kt,u}$: Intensity effect. This explains which part of the difference between the EC per capita of region *k* in year *t* and the reference region is due to the different energy efficiency of the two regions.

${D}_T^{kt,u}:$ Temperature effect. This explains which part of the difference between the EC per capita of region *k* in year *t* and the reference region is due to the different temperature of the two regions, considering the total number of days with temperatures under 0 °C and above 30 °C.

${D}_S^{kt,u}$: Structural effect. This explains which part of the difference between the EC per capita of region *k* in year *t* and the reference region is due to the different productive structure of the two regions; that is to say, the differences in the relative weight of the sectors in total production.

${D}_Y^{kt,u}$: Income effect. This explains which part of the difference between the EC per capita of region *k* in year *t* and the reference region is due to the different per capita income of the two regions.

These effects show values higher or lower than one unit depending on the role caused by the analysed variable on the per capita EC of one region compared to the average. If the analysed variable (included in one effect) increases the difference of the per capita EC of the region compared to the average, the effect shows values higher than one unit. If the variable analysed through an effect reduces the difference of the per capita EC of the region compared to the average, the effect shows values lower than one unit.

The spatial-temporal LMDI method (ST-LMDI) enables the analysis to be centred on the temporal element for a specific region. In this way, according to Ang et al. (2016), the change in the differences between the per capita EC of a region *k* and the reference region between the year *t-1* and the year *t* (SEE) is determined as follows:

6 $${SEE}^{kt-k\left(t-1\right)}=\frac{DEE^{kt-\mu}}{DEE^{k\left(t-1\right)-\mu}}=\frac{D_I^{kt,u}}{D_I^{k\left(t-1\right),u}}\cdot\frac{D_T^{kt,u}}{D_T^{k\left(t-1\right),u}}\cdot\frac{D_S^{kt,u}}{D_S^{k\left(t-1\right),u}}\cdot\frac{D_Y^{kt,u}}{D_Y^{k\left(t-1\right),u}}=S_I^{kt-k\left(t-1\right)}\cdot S_T^{kt-k\left(t-1\right)}\cdot S_S^{kt-k\left(t-1\right)}\cdot S_Y^{kt-k\left(t-1\right)}$$

Equation (6) determines the differences in the behaviour of the per capita EC of each region (AC) with respect to the reference region over time, which is explained through the intensity, temperature, structural, and income effects as follows:

${S}_I^{kt-k\left(t-1\right)}$ **:** Intensity effect. This explains which part of the change in the EC per capita of region *k* between year *t* and *t* − 1 is due to the change in the region’s energy intensity.

${S}_T^{kt-k\left(t-1\right)}:$ Temperature effect. This explains which part of the change of EC per capita of region *k* in year *t* and *t* − 1 is due to the temperature changes in the region, considering days with temperatures under 0 °C and above 30 °C.

${S}_S^{kt-k\left(t-1\right)}$: Structural effect. This explains which part of the change of EC per capita of region *k* in year *t* and *t* − 1 is due to the change in the economic structure of the region.

${S}_Y^{kt-k\left(t-1\right)}$: Income effect. This explains which part of the change in the EC per capita of region *k* between year *t t* and *t* − 1 is due to the change in the per capita income of the region.

These effects show values higher or lower than one unit depending on the role caused by the analysed variable on the per capita EC of one region compared to the average. If the variable analysed (included in one effect) increases the per capita EC of the region between period *t* and *t-1*, the effect shows values higher than one unit. If the variable analysed through an effect reduces the per capita EC of the region between period *t* and *t-1*, the effect shows values lower than one unit.

### Materials

Table 1 shows a summary statistic of the main variables used in the analysis. The analysis covers the 2000–2016 period, as these years have information for all the variables considered in the study.

**Table 1:** Stats of the main variables at the national level from 2000 to 2016

Stats	Electricity consumption (MWh)	Population (number of people)	Temperature (number of days)	GVA (constant 2010 - thousands of euros)	Income per capita (thousands of euros)
Mean	170,513,095.20	44,837,799	83.98	937,576,911.61	20.91
Min	135,338,541.00	40,358,287	60.05	789,299,564.71	19.56
Max	201,517,232.00	47,100,501	110.92	1,019,074,496.20	22.37
SD	17,515,338.43	2,307,658	12.22	68,867,088.53	0.80
1st quartile	161,988,072.24	43,055,014	79.18	896,212,259.08	20.36
2nd quartile	172,110,049.00	46,008,985	83.29	950,297,436.50	20.82
3rd quartile	177,567,692.00	46,601,869	91.99	985,038,912.61	21.18

Source: own elaboration

The data on the EC in megawatt-hours (MWh) by sector and region (see column 2 in Table 1) are taken from the Energy Statistics and Balance Sheets of the Secretary of State of Energy of the Government of Spain (Secretaría de Estado de Energía 2019).

The series of data on the population (see column 3 in Table 1) were also obtained from this institute (INE 2019a).

Two climate parameters have been used for climate correction in this analysis: the number of days per year with temperatures above 30 °C and the number of days per year with temperatures below 0 °C (see column 4 in Table 1). Both climate parameters make it possible to determine the number of days that surpass the upper and lower temperature thresholds established by AEMET (2019) for the Mediterranean climate. The calculation of these exceptional/unusual number of days with temperatures below 0 °C and above 30 °C allows to identify the changes in temperatures far from what is usual in Spain’s predominant climate. Days of extreme temperatures are analysed and recorded in the statistics of the main meteorological agencies—such as the *National Oceanic and Atmospheric Administration*, NOAA, in the USA and the *State Meteorological Agency*, AEMET, in Spain.

Globally, the climate in Spain can be divided into five main zones: a Mediterranean climate that stretches along the southern and eastern coasts up to the Pyrenees; a semiarid Mediterranean climate in the South-east; a continental Mediterranean climate in the Peninsula’s inland areas; an oceanic climate in the North-west and the coastal strip near the Bay of Biscay; and a subtropical climate in the Canary Islands (Prieto et al. 2004; AEMET 2019). The numbers of days per year with a minimum temperature lower or equal to 0 °C are shown for the provinces of León, Palencia, Lleida, Avila, Soria, Guadalajara, and Teruel, and for the Pyrenees. The lowest values are in the southern part of the Peninsula (in the Algarve, Huelva, parts of Seville and in Cadiz), in the coastal area between Almeria and Tarragona and in the Balearic Islands. The number of days in the year with a maximum temperature above or equal to 30 °C is greater than 110 days in a large part of the southern half of the Iberian Peninsula, in areas of the northeast and in the Balearic Islands.

The sectoral GVA data at constant prices with reference year 2010 expressed in euro thousands (column 5 in Table 1) originate from the statistical series of the sectoral GVA at current prices and the linked volume indices available from the Spanish National Institute of Statistics (INE 2019b). Finally, the per capita income is the GVA per capita, measured as euros by inhabitant (column 6 in Table 1).

## Results

The ST-LMDI method has been applied to analyse the key factors that explain the changes in the differences in the per capita EC of the economic sectors among the AC over the 2000–2016 period.

### Temporal analysis of the change in the EC of the AC between 2000 and 2016

**Fig. 1:**
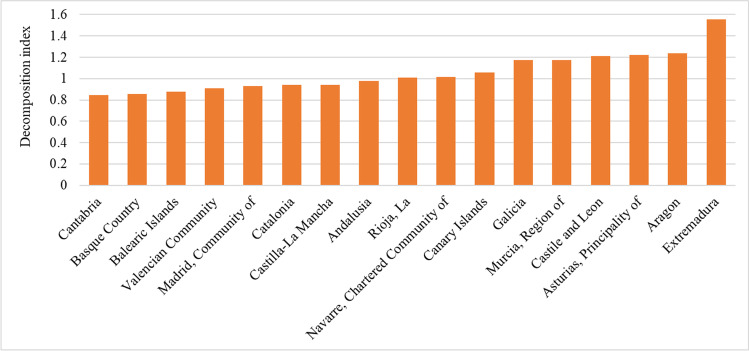
Change in the per capita EC of the AC between 2000 and 2016. *Source:* o*wn elaboration (see Table 3_column S*_*EE*_*)*

The analysis of the per capita EC of the AC over the 2000–2016 period (Fig. 1) reveals that Cantabria, the Basque Country, the Balearic Islands, the Valencian Community, the Community of Madrid, Catalonia, Castilla-La Mancha, and Andalusia reduced their per capita EC by between 2 and 16%. The case of Cantabria stands out due to the significant reduction of its per capita EC (16%), showing an average year-on-year decrease of approximately 1% throughout the period (see Table 2). The Basque Country and the Balearic Islands reduced their per capita EC by 14% and 12%, respectively, while the other AC diminished their per capita EC by between 2 and 9%.

In contrast, 9 AC increased their per capita EC between 2000 and 2016. Specifically, Extremadura registered a 55% increase over the period with a year-on-year 3% increase. Aragon, the Principality of Asturias, Castilla-La Mancha, the Region of Murcia, and Galicia registered increases ranging between 17 and 24%, while the rise in the Canary Islands, the Chartered Community of Navarre, and La Rioja was less than 6%.

Other relevant results emerge when the analysis is conducted for the two subperiods, 2000–2008 and 2008–2016 that the literature has highlighted as relevant due to the major recession which occurred in 2008 (Román-Collado and Colinet 2018a, b). This data is shown in columns S_EE_ in Table 3 in Annex B.

During the expansion period 2000–2008, all the Spanish regions increased their per capita EC (see Table 3). Extremadura recorded an increase of 71%, while the Principality of Asturias, Aragon, Galicia, the Canary Islands, and Castile and León registered a rise ranging between 27 and 43%. The rest of the AC recorded increases below 25%. As for the 2008–2016 subperiod, the results reveal that all the AC reduced their per capita EC, especially the Basque Country with a 30% decrease (see Table 3). Extremadura was the AC with the greatest fluctuations in changes of per capita EC between the two subperiods, going from a 71% increase (1.71) in the 2000–2008 subperiod to a 9% drop (0.91) between 2008 and 2016. This is a 47% decrease in the difference of the per capita EC between the two periods. The Canary Islands and the Basque Country also registered noticeable decreases by 46% and 43%, respectively, in their per capita EC. Decreases in the rest of the regions ranged from 41 to 24%.

**Fig. 2:**
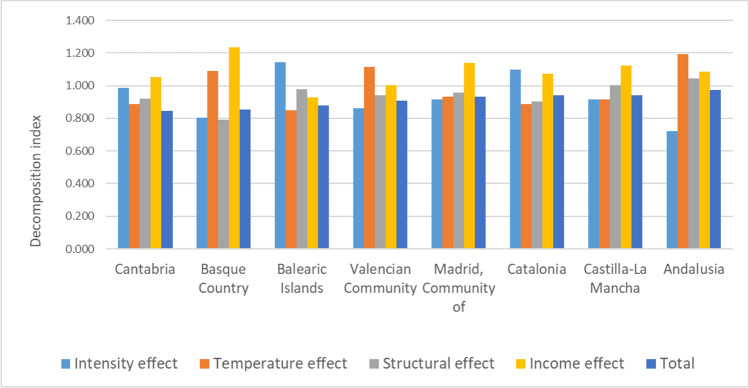
Regions with a negative per capita EC change between 2000 and 2016. Key decomposition effects. Source: own elaboration (see Table 3_columns S_I_, S_H_, S_S_, S_Y_, S_EE_)

The temporal decomposition analysis allows to identify the key effects of the changes in the per capita EC of the AC during the 2000–2016 period (see Table 3). Figure 2 shows the explanatory effects that accompanied the decreases in per capita EC for the AC between 2000 and 2016.

Figure 2 shows that with the exception of the Balearic Islands, the income effect is a driver of the per capita EC of these AC between 2000 and 2016 (the per capita GVA of these regions increased and therefore so did the per capita EC) but was completely counterbalanced by the other effects. Generally, the structural, intensity, and temperature effects act as inhibitors of the per capita EC for these AC. Specifically, apart from Andalusia and Castilla-La Mancha, the structural effect shows values below one, suggesting that there was an increase in the relative weight of lower energy intensive sectors that contributed to reduce per capita EC. The intensity effect also inhibited the per capita EC explained by an improvement in energy efficiency, except for the Balearic Islands and Catalonia. Additionally, the temperature effect also reduced per capita EC due to a decrease in temperature anomalies in 2016 compared with the year 2000, except for the Basque Country, the Valencian Community and Andalusia, where the temperature effect showed values higher than one although they are counterbalanced by the others.

**Fig. 3:**
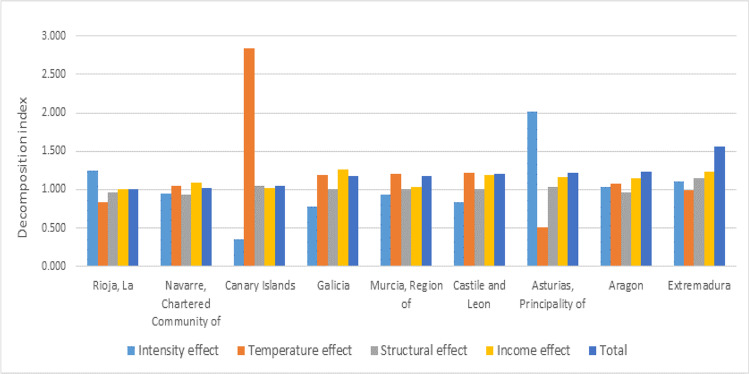
Regions with a positive per capita EC change between 2000 and 2016. Key decomposition effects. Source: own elaboration (see Table 3_columns S_I_, S_H_, S_S_, S_Y_, S_EE_)

Figure 3 depicts the information related to the regions with a positive per capita EC change between 2000 and 2016 in order to identify the main explanatory effects. Similarly to the previous group of regions, the income effect was a driver of the per capita EC in all the regions due to an increase in the per capita GVA. The regions with the highest increase in per capita EC were Aragon, Extremadura, and the Principality of Asturias. In the case of these last two AC, the driver effects were not only the income but also the structural effect.

With regard to the AC in Fig. 3, the temperature effect deserves a thorough analysis since most of the regions show values higher than one (especially in the case of the Canary Islands) except for La Rioja, the Principality of Asturias, and Extremadura. That is, the temperature effect was a driver of the per capita EC of these AC. Specifically, for these AC and the period analysed, the number of anomalous days increased and, therefore, brought about an increase in the per capita EC.

When analysing the two subperiods mentioned above, two results should be highlighted. The intensity and income effects drove the per capita EC in most of the AC during the 2000–2008 subperiod, whereas they acted as inhibitors during the 2008–2016 subperiod. On the other hand, the temperature effect shows that there were more anomalous temperatures in the 2008–2016 subperiod, mainly in the Canary Islands, which became a driver of the AC per capita EC (Table 4).

### Spatial-temporal analysis of the differences in per capita EC between the AC and the reference region

**Fig. 4:**
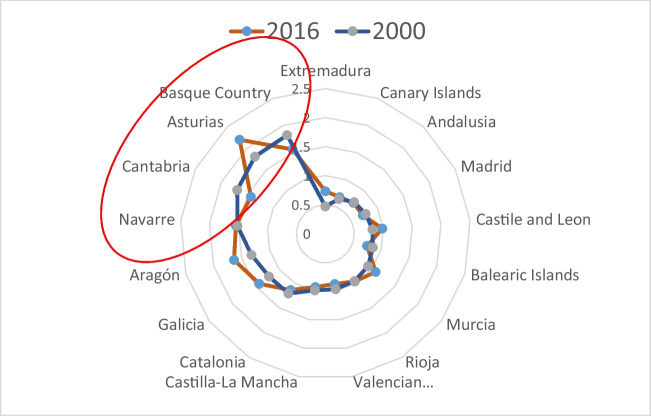
Differences in per capita EC between AC and the reference region in 2000 and 2016 (values: decomposition index). Source: own elaboration

The differences in the per capita EC of the AC compared to the reference region in 2000 and 2016 are shown in Fig. 4, where AC with values higher (lower) than one show a per capita EC above (below) the regional average. Figure 4 shows that AC closer to zero have a per capita EC similar to the average and therefore, the focus should be on the AC that reveal important differences in their per capita EC compared to the average.

The results of Fig. 4 reveal that for the year 2000, 7 AC show a per capita EC above the reference region: the Basque Country, the Principality of Asturias, Cantabria, the Chartered Community of Navarre, Aragon, Catalonia, and Galicia. These AC share certain characteristics: their geographic location (north of the country) and a higher-than-average per capita income. The case of the Basque Country stands out as having the largest positive difference in the per capita EC, 82% more than the regional average.

Conversely, 7 AC—Extremadura, the Canary Islands, Andalusia, the Community of Madrid, Castile and León, the Balearic Islands, and the Region of Murcia—recorded a per capita EC lower than the reference region. These AC all have a similar sectoral structure of their economy, based mainly on the service sector, although they are not in a similar geographic location. Extremadura and the Canary Islands registered lowest levels of per capita EC—52% and 36%, respectively—than the reference region. Finally, La Rioja, the Valencian Community, and Castilla–La Mancha recorded a similar per capita EC to that of the reference region (3.55 MWh/inhabitant).

In 2016, the behaviour of the AC was very similar to that seen in 2000, only showing small changes. Thus, the number of AC with EC levels above the reference region increased to 8, with the addition of the Region of Murcia. For this year, the Principality of Asturias and Aragon registered levels of per capita EC above the reference region, 119% and 63%, respectively. Conversely, 7 AC recorded EC levels below the average, with Extremadura being, as in 2000, the one with the lowest EC level—27% less than the reference region—while Castile and León and La Rioja had a per capita EC similar to the regional average.

Considering both years, Fig. 4 shows that the Basque Country, Principality of Asturias, the Chartered Community of Navarre (Navarre), and Cantabria stand out for showing the greatest difference in their EC per capita with respect to the average.

**Fig. 5:**
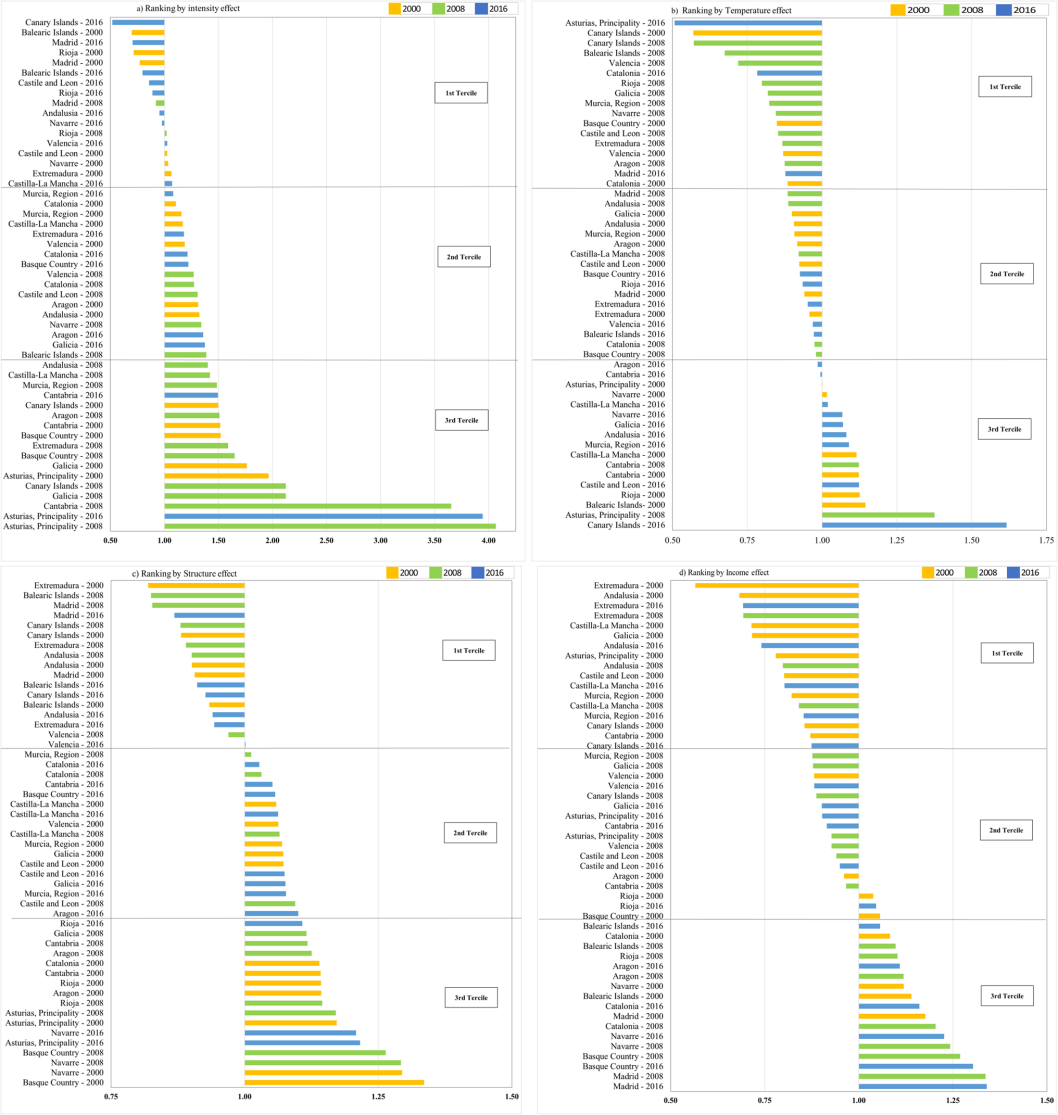
Classification of the AC by effects and differences in the per capita EC in 2000, 2008, and 2016 with respect to the reference region (values: decomposition index). Note: **a** intensity effect, **b** temperature effect, **c** structural effect, **d** per capita income effect. Source: own elaboration

In order to analyse the contributions of each analysed effect—that is, intensity, temperature, structural and income—to the differences in the per capita EC of the AC with respect to the reference region, Fig. 5 shows a classification of the AC according to the results of the effects for 2000, 2008, and 2016. Figure 5 groups the AC into three terciles depending on the significance of the analysed effect.

Figure 5a shows the contribution of the intensity effect to the differences in per capita EC of AC with respect to the average in 2000, 2008, and 2016. The results show that this effect mainly acted as a driver, considering that in most AC/years, the values are higher than one unit. The behaviour of the Canary Islands and Andalusia between 2008 and 2016 is worth noting: the intensity effect went from being a driver, increasing the per capita EC by between 265 and 40% (placing them in the third tercile in 2008) to being an inhibitor, reducing the per capita EC by between 48 and 5% (placing them in the first tercile in 2016). This change in the relative position of the Canary Islands and Andalusia between 2008 and 2016 represents an improvement of these regions’ energy efficiency in relation to the Spanish average. On the other hand, in the case of the Balearic Islands, the intensity effect went from playing an inhibitor role in 2000, reducing the per capita EC by 30% (placing this AC in the first tercile), to acting as a driver, increasing the per capita EC by 39% in 2008 (placing it in the second tercile) and therefore, worsening the position of this AC (see Tables 4, 5, 6).

Figure 5b shows the contribution of the temperature effect to the differences in per capita EC of AC with respect to the average in 2000, 2008, and 2016. The results show that this effect mainly acted as an inhibitor, considering that in most AC/years, the values are lower than unit. Seven AC recorded more days of extreme temperatures than the regional average in 2016, placing them in the third tercile. The majority of AC recorded a temperature effect lower than unit in 2000 and 2008, showing that fewer days with extreme temperatures occurred in these AC in comparison to the average. Some AC can be highlighted. The Canary Islands went from 1st position in 2000 (first tercile) to 17th in 2016 (third tercile); showing an increase in the temperature effect that caused larger differences in the per capita EC of this AC with respect to the average. The opposite occurred with the Principality of Asturias, which was 12th in 2000 (third tercile), meaning it was above the regional average, but was in 1st position in 2016 (first tercile) (see Tables 4 and 6).

Figure 5c shows the contribution of the structural effect to the differences in per capita EC of AC with respect to the average in 2000, 2008, and 2016. The results show that this effect mainly acted as a driver, considering that in most AC/years, the values are higher than one unit. It can be seen that the AC of Extremadura, the Community of Madrid, the Canary Islands, Andalusia, and the Balearic Islands have a less energy-intensive productive structure than the reference region, which contributes to placing these AC in the first tercile in 2000, 2008, and 2016. However, in spite of Extremadura having a less energy-intensive structure than the regional average in the 3 years, the productive structure has changed along the analysed period, reducing the differences in the per capita EC (see Tables 4, 5, 6). On the contrary, in the case of the AC of La Rioja, Aragon, the Principality of Asturias, and the Chartered Community of Navarre, the fact that their productive structure is more energy intensive than the regional average led to these AC being above the regional average in 2000, 2008, and 2016 (in the third tercile). The Basque Country experienced changes in its productive structure throughout the period, reducing the positive differences in EC with respect to the reference region; as a result, it went from the third tercile (1.33) in 2000 to the second (1.06) in 2016 (Table 7).

Figure 5d shows the contribution of the income effect to the differences in per capita EC of AC with respect to the average in 2000, 2008, and 2016. The results show that this effect mainly acted as an inhibitor, considering that in most AC/years, the values are lower than one unit. The results of the per capita income effect indicate an important territorial disparity. In the case of Extremadura, Andalusia, and Castilla-La Mancha (in the first tercile), the per capita income effect contributed to their per capita EC being between 16 and 44% below the regional average over the entire period. On the contrary, in the Basque Country, the Community of Madrid, the Balearic Islands, Catalonia, the Chartered Community of Navarre, and La Rioja, the income effect contributed to an increase in the per capita EC of between 4 and 34%, placing them above the regional average throughout the period (third tercile).

## Discussion

The literature review shows evidence of the nexus between energy consumption and economic growth (Li and Leung 2021; García-Amate and Ramírez-Orellana 2021) but there is limited evidence of the nexus with the EC of economic sectors. The results of the ST-LMDI analysis have led to identifying that the income effect is a driver of the per capita EC of all AC throughout the analysed period. An additional result is that the final per capita EC growth has been decoupled from per capita income growth in AC such as Cantabria, the Basque Country, the Valencian Community, Madrid, Catalonia, Castilla-La Mancha, and Andalusia. This is an important issue that has been widely analysed for China and other Asian countries, but little evidence has been shown for Europe or Spain (Zhang et al. 2019b; Lin and Raza 2021; Zheng et al. 2022; Moutinho et al. 2018; Zhang and Wang 2013), and would require more attention for researchers.

The data for the structural effect show that there have not been great changes in the relative weight of economic sectors in the AC, although in some were notable such as Extremadura and the Basque Country where the per capita EC increased by 15% and decreased by 31%, respectively, due to the changes in the sectors’ structure. The actual decarbonisation of the economies promotes the use of cleaner energy sources and of course, cleaner energy production processes such as electricity (Tol 2021). Therefore, this trend must be considered when analysing the changes in Spanish EC. The change in EC due to the structural effect must be analysed in detail. For example, a decrease in EC might be due to the more energy-intensive sectors such as the industrial sector which has reduced its weight on total economy or to the fact that the energy use of the economic sectors is different from that of electricity (Veselov et al. 2021; Madeddu et al. 2021). This is what has happened in the Basque economy, which became less intensive in EC at the end of the period, as a result of the loss of the relative weight of industrial sector in the GDP (only representing 21.8% of the total output in 2016) (Instituto Vasco de Estadística 2018). The contrary has happened in Extremadura, where the increase in per capita EC is also due to an important growth of per capita GVA (3.2%), the more electricity-intensive economic sectors (Romero et al. 2019; Pérez-García and Moral Carcedo 2017).

Spain, along with Germany, France, and Italy, is among the European countries that have a higher energy consumption demand, accounting for over 56% of the final energy consumption (Tsemekidi Tzeiranaki et al. 2022). However, the decoupling process between energy consumption and economic growth has allowed to reduce energy intensity in Spain throughout the analysed period, although it is more relevant in the second subperiod between 2008 and 2016 (METAD 2016; REE 2019). The results of the analysis conducted on the intensity effect show that although most AC show energy efficiency improvements between 2000 and 2016, this did not occur in certain AC such as the Balearic Islands, Catalonia, La Rioja, Asturias, Aragon, and Extremadura. This result is worse for AC such as Catalonia, Asturias, and Aragon because their energy intensity was greater than the average and this did not decrease throughout the period.

Focussing on the AC registering positive differences in their per capita EC with respect to the regional average (the Basque Country, Cantabria, the Principality of Asturias, the Chartered Community of Navarre, Aragon, Galicia, and Catalonia), they share certain characteristics. Firstly, the manufacturing industrial sector of these AC accounts for a greater share of their GVA (more intensive in energy); that is to say, the weight of the industry is greater in these regions by 14 percentage points with respect to the reference region. And secondly, in the Basque Country, the Chartered Community of Navarre and Catalonia, the per capita income level is 31% higher than the regional average. Based on these differentiating features and considering the results obtained in the decomposition analysis, it is noted that the per capita income of these AC is higher than the average, allowing them to reduce their per capita EC (except the Basque Country, the Chartered Community of Navarre, and Catalonia) by 28% in 2000 and 10% in 2016, and the intensity and structural effects explain these AC exceeding the per capita EC regional average.

The results of the temperature effect in Spain show that more days with extreme temperatures were recorded during the second part of the analysed period (2008–2016) compared to the first one (2000–2009). Specifically, 14 out of 17 AC increased their per capita EC due to an increase in the number of days with extreme temperatures. This result is in line with previous research that have highlighted that the records in recent decades showed abnormally high temperatures during the summer, especially southern Europe (Luterbacher et al. 2016) and indicate significant warming in the Spanish Mediterranean region (Quereda et al. 2020). Additionally, the characteristic adverse effects of climate change (Ma and Zhu, 2019) also include severe one-off drops in temperature. A recent example of this is Storm Filomena in 2021, which brought temperatures far below the average minimum temperatures in Spain. On 12th January, there was a record minimum temperature of −26.5 °C (in Teruel, in the AC of Aragon) and −25.3 °C (in Guadalajara, in the AC of Castilla-La Mancha) (AEMET 2021).

The results of the decomposition analysis show that temperature changes resulting from global warming cause variations in the levels of EC in the economic sectors (Wenz et al. 2017; Xu and Ang 2014). As for Spain, previous research indicates that global warming may induce a growth in electricity demand in Spain, especially related to cooling needs (Pablo-Romero et al. 2022). This paper shows that between 2000 and 2016, there was an increase in the number of days with extreme temperatures that led to an increase ranging between 5 and 184% in the per capita EC of some Mediterranean AC (Andalusia, Murcia, and the Valencian Community) with a subtropical climate (the Canary Islands), continental climate (Castile and León, Aragon, and Navarre) and Oceanic climate (the Basque Country and Galicia).

Andalusia, Murcia, the Valencian Community, the Canary Islands, and Galicia are AC where the service sector is highly representative above other sectors. So, the extreme temperatures, as it was mentioned before, are increasing the EC coming from all economic sectors but particularly, from the service sector. Literature shows that the weather and climate conditions are identified as important influencing factors in the tertiary energy consumption as comfort conditions play a key role within the sector (Tsemekidi Tzeiranaki et al. 2022). Furthermore, outdoor activities for tourists are also be affected by the weather conditions, as they are highly depended on climate and thermal comfort (Karimi and Mohammad 2022; Cardell et al. 2022). This is particularly relevant in the case of Andalusia and the Canary Islands where the tourism sector represents 13% and 34% of GVA, respectively. Since the competitiveness of these AC would be affected by future changes in weather conditions, it should be considered by the national and regional governments. More efforts should be made to promote reducing EC in the service sector, which require focussing on lighting, appliances, ICT, and more efficient heating and cooling equipment.

The demand for electricity used for heating and cooling spaces is also relevant in the industry sector, which combines demand for heating and cooling for industrial processes and for spaces (Rehfeldt et al. 2018; Persson et al. 2015). Additionally, the service sector and specifically, the commercial and tourism sectors have a high demand of heating and cooling systems. This demand is quite relevant in countries that have high temperatures over long periods but also in other countries that only have high temperatures over short periods, because in this later case, households rarely invest on heating and cooling systems but large offices and commercial building do (IEA 2018). Therefore, action must be taken to reduce the impact of these changes in extreme temperatures on EC in the Spanish AC. Measures such as the Energy Rehabilitation Programme for Buildings (MITECO 2020) represent a step towards the thermal insulation of buildings, contributing to reducing EC resulting not only from the temperature effect but also from the intensity effect, thus resulting in efficiency gains.

## Conclusions and policy implications

The results reveal that the extreme changes of temperature in Spain between 2000 and 2016 have substantially affected the per capita EC of Spanish AC. Likewise, it has been noted that in the 2000–2008 subperiod, the temperature effect mainly acted as an inhibitor, unlike in the 2008–2016 subperiod. The temporal decomposition analysis shows that the extreme temperatures were mostly concentrated in the second subperiod causing an increase in EC per capita of AC such as Navarre, Galicia, Murcia, Castile and León, and the Canary Islands. This trend should be considered for two reasons. First, the extreme temperatures are concentrated in the last part of the period analysed, so future temperature changes should be considered. Second, the AC that are most affected are those where the economic activity is highly dependent on the industry sector (Navarre and Castile and León) or on the service sector (Canary Islands, Galicia, Murcia). So, if the aim is to prevent the temperature effect in the future, EC should be decoupled from temperatures.

The spatial decomposition analysis conducted shows important results in what regards economic sectors, temperatures, and EC. AC in the north of Spain, such as the Basque Country, Cantabria, the Principality of Asturias, the Chartered Community of Navarre, Galicia, Aragon, and Catalonia, characterised by the predominance of the industrial sector and a high per capita income have reached a per capita EC surpassing the regional average in 2000, 2008, and 2016. In these same AC, the temperature effect has acted as an inhibitor, helping to reduce the differences in per capita EC with respect to the regional average, mainly in 2008 and 2016.

On the other hand, the AC in the south of Spain (Extremadura and Andalusia) and the Canary Islands, which have a less energy-intensive productive structure and a lower per capita income, shows a per capita EC below the reference region in 2000, 2008, and 2016. Furthermore, the less efficient use of EC in these AC has given rise to the per capita EC due to the intensity effect being higher than the regional average in 2000 and 2008. Finally, the extreme temperatures in 2016 increased the per capita EC differences in these AC with respect to the reference region, although they were offset by the other effects, meaning that in the end, the per capita EC of these AC was below the regional average.

The results of this analysis show that temperature is one of the key factors in the final EC of AC. On the one hand, the mild temperatures in the North have helped to partially offset the high EC of the AC in this area, characterised by energy-intensive economic sectors. On the other hand, the high temperatures in the South have raised the EC of these AC, characterised by less energy-intensive economic sectors. These results are conditioned by the choice of limit values for extreme temperatures, selecting a value greater than 30 °C (for the calculation of days with extreme high temperatures) and a value less than 0 °C (for the calculation of days with extreme low temperatures) in Spain. However, although this selection of temperatures is restrictive, in general, the results are in line with the research literature, showing a significant warming of the Spanish Mediterranean regions and a severe drop in temperatures in the north, which has led to an increase in the number of days of what we have identified as extreme temperatures.

To sum up, it should be considered that future temperature increases might cause severe inefficiencies in the AC if measures have not been implemented to reduce the temperature effect on EC.

Therefore, the regional environmental regulation should be focused on elements that contribute to reduce the impact of extreme temperatures in EC of the AC, and therefore, in the economic sectors, i.e., promoting savings in lighting, ICT, and heating and cooling appliances. Additionally, building insulation should be improved, not only in the commercial and tourist sector but also in the Public Administration and industrial offices.

## Data Availability

The datasets generated and/or analysed during the current study are available from the corresponding author on reasonable request.

## ANNEX A

According to Ang et al. (2015), the effects can be calculated as follows:

A.1 $${D}_I^{kt,u}=\mathit{\exp}\left(\sum_i^n{w}_{ik,\mu}\cdot \ln \left(\frac{I_i^{kt}}{I_i^u}\right)\right)$$

A.2 $${D}_T^{kt,u}=\mathit{\exp}\left(\sum_i^n{w}_{ik,\mu}\cdot \ln \left(\frac{T^{kt}}{T^u}\right)\right)$$

A.3 $${D}_S^{kt,u}=\mathit{\exp}\left(\sum_i^n{w}_{ik,\mu}\cdot \ln \left(\frac{S_i^{kt}}{S_i^u}\right)\right)$$

A.4 $${D}_Y^{kt,u}=\mathit{\exp}\left(\sum_i^n{w}_{ik,\mu}\cdot \ln \left(\frac{Y^{kt}}{Y^u}\right)\right)$$

The weighting factor *w*_*ik*, *μ*_ is the logarithmic average of the per capita EC of sector *i* between region *k* and the reference region *μ*, with respect to the logarithmic average of the total per capita EC between region *k* and the reference region *μ*. The weighting factor is calculated as follows:

A.5 $${w}_{ik,\mu }=\frac{\frac{\frac{EE_i^{kt}}{P^t}-\frac{EE_i^{\mu }}{P^{\mu }}}{\ln \left(\frac{EE_i^{kt}}{P^t}\right)-\ln \left(\ \frac{EE_i^{\mu }}{P^{\mu }}\right)}}{\frac{{EE pc}^{kt}-{ EE pc}^{\mu }}{\ln \left({ EE pc}^{kt}\right)-\ln \left({ EE pc}^{\mu}\right)}}$$

## ANNEX B

**Table 2:** The interannual change of the per capita EC of AC between 2000 and 2016*

CCAA	2000–2001	2001–2002	2002–2003	2003–2004	2004–2005	2005–2006	2006–2007	2007–2008	2008–200**9**	2009–2010	2010–201**1**	2011–2012	2012–201**3**	2013–2014	2014–2015	2015–2016
Andalusia	0.928	1.024	1.098	1.016	1.005	1.108	1.027	0.975	0.859	0.987	0.971	1.014	0.937	1.001	1.044	1.010
Aragón	1.094	0.987	1.112	1.007	1.003	1.056	1.029	0.996	0.883	1.010	1.009	0.998	0.945	1.013	1.046	1.049
Asturias, Principality	1.086	1.090	1.023	0.871	0.531	2.092	1.010	1.076	0.916	0.990	1.108	0.968	0.918	1.044	1.052	0.975
Balearic Islands	1.030	0.981	1.045	1.023	0.690	1.517	0.984	0.981	0.887	0.932	0.975	1.008	0.962	0.987	1.080	0.965
Canary Islands	1.048	1.002	1.087	1.056	0.993	1.106	1.004	1.053	0.907	0.860	0.935	1.028	0.979	1.015	1.042	0.971
Cantabria	1.028	0.963	1.071	1.029	0.735	1.372	0.989	0.991	0.838	1.118	1.092	0.986	0.914	1.005	0.935	0.902
Castile and León	1.055	1.068	1.065	1.051	1.037	1.013	1.062	1.016	0.919	0.919	0.956	1.021	0.987	1.016	1.375	0.744
Castilla-La Mancha	1.069	1.010	1.079	1.025	1.095	0.961	0.995	0.955	0.960	0.898	0.973	1.064	0.900	1.018	0.994	0.969
Catalonia	1.152	1.000	1.029	1.005	0.525	1.957	1.013	0.970	0.880	0.958	0.981	0.986	0.937	0.992	1.073	0.960
Community Valencia	1.046	1.023	1.028	1.029	0.728	1.416	1.013	0.940	0.888	0.955	0.948	1.001	0.985	1.024	1.028	0.977
Extremadura	1.034	1.060	1.105	1.055	1.144	1.063	1.068	1.028	0.975	0.992	0.934	1.012	0.889	0.958	1.082	1.083
Galicia	1.066	0.958	1.122	1.042	0.662	1.599	1.035	1.011	0.924	0.978	0.990	1.026	0.959	1.010	0.997	0.999
Madrid, Community of	1.027	1.021	1.040	1.031	0.461	2.275	1.016	1.025	0.913	0.935	1.018	0.943	0.909	0.973	1.050	0.996
Murcia, Region	1.053	1.032	1.065	1.005	0.827	1.246	1.035	1.003	0.896	0.907	0.982	1.023	1.093	1.021	1.060	0.978
Navarre, Chartered Community	1.030	1.078	1.012	1.041	1.029	0.985	1.041	0.977	0.875	0.971	0.996	0.993	0.971	1.029	0.985	1.021
Basque Country	1.042	1.030	1.020	1.071	0.730	1.404	1.023	1.000	0.772	1.008	0.975	0.977	0.899	1.083	1.031	0.936
Rioja, La	1.036	1.020	1.047	1.032	0.996	1.011	1.060	1.006	0.887	0.968	0.922	0.946	1.075	1.035	0.973	1.013

Source: own elaboration. *These data originate from the application of Eq. 6. The data allows us to identify how the per capita EC of each AC behaves throughout the analysed period. If the data is higher than one unit, it means that the per capita EC has increased, and if the data shows a value lower than one unit, it means that the per capita EC has decreased throughout the analysed period

**Table 3:** Decomposition effects of the per capita EE change between 2000 and 2016*

AC	S _I_	S_T_	S _S_	S _Y_	S _EE_
Cantabria	0.986	0.885	0.921	1.051	0.844
Basque Country	0.803	1.090	0.791	1.234	0.855
Balearic Islands	1.144	0.850	0.976	0.927	0.879
Valencian Community	0.863	1.114	0.942	1.001	0.907
Madrid, Community of	0.914	0.933	0.958	1.139	0.930
Catalonia	1.098	0.886	0.901	1.072	0.940
Castilla-La Mancha	0.914	0.914	1.003	1.123	0.941
Andalusia	0.722	1.194	1.043	1.086	0.975
Rioja, La	1.244	0.830	0.969	1.007	1.008
Navarre, Chartered Community of	0.946	1.051	0.934	1.096	1.017
Canary Islands	0.346	2.836	1.052	1.022	1.055
Galicia	0.779	1.191	1.004	1.259	1.173
Murcia, Region of	0.935	1.201	1.007	1.039	1.175
Castile and León	0.837	1.217	1.002	1.185	1.209
Asturias, Principality of	2.009	0.506	1.037	1.158	1.220
Aragon	1.037	1.074	0.963	1.154	1.237
Extremadura	1.108	0.994	1.151	1.227	1.555

Source: own elaboration. *These data originate from the application of Eq. 6. Data should be interpreted as follows. If the data is higher than one unit, it means that the per capita EC has increased, and if the data shows a value lower than one unit, it means that the per capita EC has decreased throughout the analysed period. Note: S_I_, intensity effect; S_T_, temperature effect; S_S_, structural effect; S_Y_, per capita income. Column S_EE shows the per capita EC change of each AC between 2000 and 2016. Column S_I shows the per capita EC change resulting from a change in the AC’s energy intensity. Column S_T shows the per capita EC change resulting from a change in the AC’s temperatures. Column S_S shows the per capita EC change resulting from a change in the AC’s economic structure. Column S_Y shows the per capita EC change resulting from a change in the AC’s per capita income

**Table 4:** Decomposition effects of the per capita EE change between 2000–2008 and 2008–2016

AC	2000*–*2008					2008*–*2016				
	S _I_	S _T_	S _S_	S _Y_	S _EE_	S _I_	S _T_	S _S_	S _Y_	S _EE_
Asturias, Principality of	2.069	0.516	0.999	1.196	1.274	0.971	0.980	1.039	0.968	0.957
Murcia, Region of	1.282	0.966	0.946	1.062	1.244	0.729	1.244	1.064	0.979	0.945
Aragon	1.150	1.009	0.985	1.150	1.312	0.902	1.065	0.978	1.004	0.943
Extremadura	1.490	0.834	1.086	1.263	1.706	0.743	1.192	1.059	0.971	0.911
Galicia	1.204	0.844	1.041	1.251	1.322	0.648	1.411	0.965	1.006	0.887
Castile and León	1.272	0.945	1.020	1.166	1.431	0.658	1.287	0.982	1.016	0.845
Navarre, Chartered Community of	1.295	0.847	0.998	1.100	1.205	0.730	1.241	0.935	0.996	0.844
Andalusia	1.059	0.958	1.000	1.166	1.183	0.681	1.246	1.043	0.931	0.825
Rioja, La	1.425	0.823	1.002	1.044	1.228	0.873	1.008	0.967	0.965	0.821
Valencian Community	1.068	1.105	0.912	1.032	1.110	0.808	1.008	1.033	0.970	0.817
Balearic Islands	1.989	0.665	0.883	0.936	1.092	0.575	1.279	1.105	0.990	0.805
Castilla-La Mancha	1.212	0.835	1.006	1.174	1.194	0.754	1.095	0.997	0.957	0.788
Cantabria	2.407	0.416	0.978	1.100	1.079	0.409	2.126	0.942	0.955	0.783
Catalonia	1.150	1.064	0.905	1.087	1.204	0.955	0.832	0.996	0.986	0.781
Madrid, Community of	1.194	1.017	0.912	1.109	1.229	0.766	0.917	1.050	1.026	0.757
Canary Islands	1.417	0.974	0.999	1.017	1.401	0.244	2.913	1.053	1.004	0.753
Basque Country	1.085	0.993	0.946	1.206	1.229	0.741	1.098	0.836	1.023	0.696

Source: own elaboration. *These data come from the application of Eq. 6. Data should be interpreted as follows. If data is higher than one unit, it means that the per capita EC has increased and if data shows a value lower than one unit, it means that the per capita EC has been reduced along the period analysed. Note: S_I_, intensity effect; S_T_, temperature effect; S_S_, structural effect; S_Y_, per capita income. Column S_EE shows the per capita EC change of each AC between 2000 and 2016. Column S_I shows the per capita EC change resulting from a change in the AC’s energy intensity. Column S_T shows the per capita EC change resulting from a change in the AC’s temperatures. Column S_S shows the per capita EC change resulting from a change in the AC’s economic structure. Column S_Y shows the per capita EC change resulting from a change in the AC’s per capita income

**Table 5:** Classification of the AC due to the per capita EE differences with respect to the average. Year 2000

AC	Ranking group AC - 2000				
	D _I_	D _T_	D _S_	D _Y_	D _EE_
Andalusia	12	6	3	2	3
Aragon	11	8	14	11	13
Asturias, Principality of	17	12	15	5	16
Balearic Islands	1	17	5	16	6
Canary Islands	13	1	2	8	2
Cantabria	14	15	12	9	15
Castilla-La Mancha	4	9	10	6	5
Castile and León	9	14	6	3	10
Catalonia	7	4	11	14	11
Valencian Community	10	3	7	10	9
Extremadura	6	11	1	1	1
Galicia	16	5	9	4	12
Madrid, Community of	3	10	4	17	4
Murcia, Region of	8	7	8	7	7
Navarre, Chartered Community of	5	13	16	15	14
Basque Country	15	2	17	13	17
Rioja, La	2	16	13	12	8

Source: own elaboration. *These data originate from the application of Eq. 5. Data should be interpreted as follows. The columns show data ranging from 1 to 17. The AC with smaller per capita EC differences with respect to the average will have lower numbers, while AC that have greater per capita EC differences with respect to the average will have higher numbers. Note: *D*_I_, intensity effect; *D*_T_, temperature effect; D_S_, structural effect; D_Y_, per capita income. D_I shows the AC ranked according to their differences in electricity per capita with respect to the average due to the intensity effect. D_T shows the AC ranked according to their differences in electricity per capita with respect to the average due to the temperature effect. D_S shows the AC ranked according to their differences in electricity per capita with respect to the average due to the structural effect. D_Y shows the AC ranked according to their differences in electricity per capita with respect to the average due to the per capita income effect. D_EE shows the AC ranked according to their total differences in electricity per capita with respect to the average

**Table 6:** Classification of the AC according to the per capita EE differences with respect to the average. Year 2008

AC	Ranking group AC - 2008				
	D _I_	D _T_	D _S_	D _Y_	D _EE_
Andalusia	8	9	5	2	2
Aragon	11	12	13	13	13
Asturias, Principality of	17	2	15	8	17
Balearic Islands	7	5	1	11	4
Canary Islands	14	3	3	4	3
Cantabria	16	1	12	10	14
Castilla-La Mancha	5	10	10	9	8
Castile and León	9	14	9	3	10
Catalonia	4	15	8	14	11
Valencian Community	3	17	6	7	6
Extremadura	12	6	4	1	1
Galicia	15	4	11	6	12
Madrid, Community of	1	16	2	17	5
Murcia, Region of	10	11	7	5	7
Navarre, Chartered Community of	6	8	17	15	15
Basque Country	13	7	16	16	16
Rioja, La	2	13	14	12	9

Source: own elaboration. *These data originate from the application of Eq. 5. Data should be interpreted as follows. The columns show data ranging from 1 to 17. The AC that have smaller per capita EC differences with respect to the average will show lower numbers, while AC with greater per capita EC differences with respect to the average will show higher numbers. Note: *D*_I_, intensity effect; *D*_T_, temperature effect; D_S_, structural effect; D_Y_, per capita income. D_I shows the AC ranked according to their differences in electricity per capita with respect to the average due to the intensity effect. D_T shows the AC ranked according to their differences in electricity per capita with respect to the average due to the temperature effect. D_S shows the AC ranked according to their differences in electricity per capita with respect to the average due to the structural effect. D_Y shows the AC ranked according to their differences in electricity per capita with respect to the average due to the per capita income effect. D_EE shows the AC ranked according to their total differences in electricity per capita with respect to the average

**Table 7:** Classification of AC according to their per capita EE differences with respect to the average. Year 2016

AC	Ranking Group AC - 2016				
	D _I_	D _T_	D _S_	D _Y_	D _EE_
Andalusia	6	14	4	2	2
Aragon	14	9	14	13	16
Asturias, Principality of	17	1	17	8	17
Balearic Islands	3	8	2	12	5
Canary Islands	1	17	3	5	1
Cantabria	16	10	8	9	13
Castilla-La Mancha	4	16	11	10	9
Castile and León	9	11	10	3	7
Catalonia	12	2	7	14	11
Valencian Community	8	7	6	6	6
Extremadura	11	6	5	1	4
Galicia	15	13	12	7	12
Madrid, Community of	2	3	1	17	3
Murcia, Region of	10	15	13	4	10
Navarre, Chartered Community of	7	12	16	15	14
Basque Country	13	4	9	16	15
Rioja, La	5	5	15	11	8

Source: own elaboration. *These data originate from the application of Eq. 5. Data should be interpreted as follows. The columns show data ranging from 1 to 17. The AC that show smaller per capita EC differences with respect to the average will show lower numbers, while AC with greater per capita EC differences with respect to the average will show higher numbers. Note: *D*_I_, intensity effect; *D*_T_, temperature effect; D_S_, structural effect; D_Y_, per capita income. D_I shows the AC ranked according to their differences in electricity per capita with respect to the average due to the intensity effect. D_T shows the AC ranked according to their differences in electricity per capita with respect to the average due to the temperature effect. D_S shows the AC ranked according to their differences in electricity per capita with respect to the average due to the structural effect. D_Y shows the AC ranked according to their differences in electricity per capita with respect to the average due to the per capita income effect. D_EE shows the AC ranked according to their total differences in electricity per capita with respect to the average

## Acronyms

*AC*: Autonomous Communities
*EC*: Electricity consumption
*ODEX*: Energy efficiency index used in the ODYSSEE-MURE project
*GVA*: Gross value added
*IDA*: Index decomposition analysis
*ICT*: Information and communication technology
*LMDI*: Logarithmic mean Divisia index
*MWh*: Megawatt-hours
*SDA*: Structural decomposition analysis
*ST-IDA*: Spatial-temporal index decomposition analysis
*ST-LMDI*: Spatial-temporal index decomposition analysis through logarithmic mean Divisia index
